# Structurally Colored Radiative Cooling Cellulosic Films

**DOI:** 10.1002/advs.202202061

**Published:** 2022-07-17

**Authors:** Wenkai Zhu, Benjamin Droguet, Qingchen Shen, Yun Zhang, Thomas G. Parton, Xiwei Shan, Richard M. Parker, Michael F. L. De Volder, Tao Deng, Silvia Vignolini, Tian Li

**Affiliations:** ^1^ School of Mechanical Engineering Purdue University West Lafayette IN 47906 USA; ^2^ Yusuf Hamied Department of Chemistry University of Cambridge Cambridge CB2 1EW UK; ^3^ State Key Laboratory of Metal Matrix Composites School of Materials Science and Engineering Shanghai Jiao Tong University Shanghai 200240 P. R. China; ^4^ Department of Engineering University of Cambridge Cambridge CB2 1PZ UK

**Keywords:** cellulose, roll‐to‐roll deposition, structural color, sub‐ambient radiative cooling, sustainability

## Abstract

Daytime radiative cooling (DRC) materials offer a sustainable approach to thermal management by exploiting net positive heat transfer to deep space. While such materials typically have a white or mirror‐like appearance to maximize solar reflection, extending the palette of available colors is required to promote their real‐world utilization. However, the incorporation of conventional absorption‐based colorants inevitably leads to solar heating, which counteracts any radiative cooling effect. In this work, efficient sub‐ambient DRC (Day: −4 °C, Night: −11 °C) from a vibrant, structurally colored film prepared from naturally derived cellulose nanocrystals (CNCs), is instead demonstrated. Arising from the underlying photonic nanostructure, the film selectively reflects visible light resulting in intense, fade‐resistant coloration, while maintaining a low solar absorption (≈3%). Additionally, a high emission within the mid‐infrared atmospheric window (>90%) allows for significant radiative heat loss. By coating such CNC films onto a highly scattering, porous ethylcellulose (EC) base layer, any sunlight that penetrates the CNC layer is backscattered by the EC layer below, achieving broadband solar reflection and vibrant structural color simultaneously. Finally, scalable manufacturing using a commercially relevant roll‐to‐roll process validates the potential to produce such colored radiative cooling materials at a large scale from a low‐cost and sustainable feedstock.

## Graphical abstract

Structurally colored films capable of full‐time sub‐ambient radiative cooling are prepared from a composite of cellulose nanocrystals and ethylcellulose. The distinct nanostructure within each layer allows for selective reflection of visible light generating color, whilst maintaining a broadband solar reflection. Finally, by demonstrating roll‐to‐roll fabrication, the potential for commercial production from this low‐cost and sustainable feedstock is substantiated.

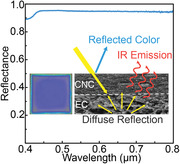

## 1 Introduction

Thermal management is crucial across all length scales, from vehicles to buildings and power plants, and is intimately related to global warming. As an indispensable aspect, cooling is often achieved by compression‐based systems (such as for air conditioning), which consume an enormous amount of energy. Guided by the concept of harvesting the coldness of the universe through passive radiative heat transfer, daytime radiative cooling (DRC) materials and technologies have proliferated over the last decade. This includes selective infrared (IR) emission by dielectric materials^[^ ^1^ ^]^ and broadband IR emission by polymer‐based materials,^[^ ^2^ ^]^ where state‐of‐the‐art designs can lower the temperature below the freezing point of the ambient environment and can provide more than 100 W m^−2^ cooling power without any energy input.^[^ ^3^ ^]^

Most materials proposed for DRC are designed to maximize reflection across the entire solar spectrum, including the visible range. Therefore, such materials tend to have a white or mirror‐like appearance, depending on whether the reflection is diffusive or specular. This limited color palette is a drawback, particularly in applications where visual aesthetics are a key consideration, such as for architecture and in consumer‐oriented industries. However, the incorporation of conventional colorants, such as dyes and pigments, into DRC materials results in high absorptance across the solar spectrum, arising from both the necessary absorption for visible color generation and additional absorptions in the near‐IR (NIR) band.^[^ ^4^ ^]^ As such, the heating associated with the absorption from dyes and pigments counteracts the radiative cooling effect from such materials. To date, the main strategy to overcome this issue has focused on lowering unnecessary solar absorption and increasing emissivity at the atmospheric transparent window (*λ* = 8–13 µm).^[^ ^5^ ^]^ For example, increasing the reflectivity in the NIR can lower the temperature relative to a colored coating or paint, but did not achieve sub‐ambient cooling.^[^ ^4^, ^6^ ^]^ Alternatively, metal–dielectric–metal or Tamm resonance structures can be combined into multilayered selective emitters to realize narrowband optical absorption, allowing for both subtractive colors and daytime sub‐ambient cooling.^[^ ^7^ ^]^ However, such top‐down layer‐by‐layer deposition processes require highly accurate control over the thickness, which hinders the realization of large‐scale photonic structures. It has also been recently reported that photoluminescent dyes, which can partially convert ultraviolet light into visible light, can be used for coloration while reducing the light–heat conversion in the solar spectral range.^[^ ^7a,b^ ^]^ However, their color intensity and cooling performance are strongly dependent on the quantum yield of the dye.

To avoid this issue of absorption‐based heating, structural coloration (where light is selectively reflected by a periodic nanostructure) can instead be considered. Such an approach to colored DRC has been explored using a silica opal structure, however sub‐ambient cooling was not achieved.^[^ ^8^ ^]^ In addition, such opal‐like photonic structures rely on the use of extremely monodisperse nanoparticles, which are challenging to produce cost‐effectively at scale. A scalable and sustainable alternative for structural coloration can be achieved with cellulose nanocrystals (CNCs). These colloidal nanoparticles are extracted from renewable sources including cotton and wood pulp.^[^ ^9^ ^]^ Once extracted, CNCs can be dispersed in water where they can spontaneously self‐organize into a colloidal liquid crystal with cholesteric (i.e., chiral nematic) order. Upon evaporation, this left‐handed cholesteric ordering can be maintained into the solid state. The helicoidal arrangement of the birefringent CNC nano‐rods in such a film results in a periodic variation in the refractive index of the nanostructure, giving rise to an intense reflection of visible light. The specific color is related to the periodicity of the helicoidal structure (known as the pitch) and is tunable across the visible spectrum.^[^ ^10^ ^]^ Importantly, it was recently shown that such films can be produced at scale from a commercial CNC source using a continuous deposition process.^[^ ^11^ ^]^

**Figure 1:**
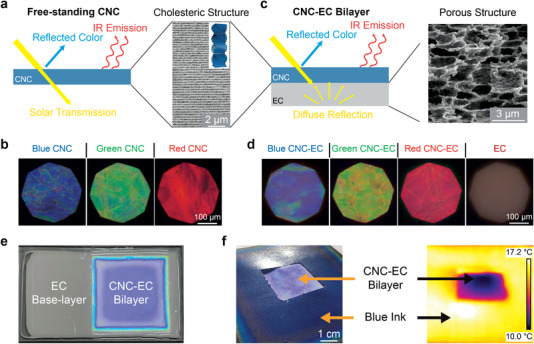
Colored radiative cooling from photonic cellulosic films. a) Schematic of the radiative energy transport in a colored and free‐standing CNC film, and cross‐sectional scanning electron microscopy (SEM) image of the cholesteric structure of the self‐assembled CNC film. b) Left‐circularly polarized (LCP) optical micrographs of the blue, green, and red CNC films. c) Schematic of the radiative energy transport and light interaction within the CNC–EC bilayer film, and cross‐sectional SEM image of the porous EC layer. d) LCP optical micrographs of the blue, green, and red CNC–EC bilayer films, with a white EC film for reference. e) Macroscopic image of an EC substrate half coated with a blue CNC photonic film. f) Digital and thermal images of a blue CNC–EC bilayer film partially overlaid with an ink‐printed film with a hue matched to the specular color of the CNC. The thermal image shows the blue CNC–EC bilayer film has a lower temperature.

In this work, we demonstrate DRC from structurally colored cellulosic films with sub‐ambient surface temperatures. First, we demonstrate that photonic CNC films combine the inherent properties of cellulose (i.e., strong mid‐IR emittance and negligible absorption across the solar range) with the wavelength‐selective reflection of the cholesteric nanostructure, with the response tunable across the entire visible range (**Figure** **1**a,b). Second, by self‐assembling a CNC layer on top of a porous ethylcellulose (EC) layer, we achieve a colored DRC cellulosic bilayer film with low solar transmission (Figure 1c,d). This allows for the colored specular reflection of the CNC layer to be coupled to the broadband diffusive response of the EC layer (Figure 1e,f). Finally, to validate the potential for commercial manufacturing, we use a roll‐to‐roll (R2R) deposition process to produce the structurally colored cellulosic DRC films at the meter‐scale.

### 1.1 Daytime Radiative Cooling Using Structurally Colored CNC Films

Structurally colored films were prepared from a commercial CNC source (University of Maine Process Development Center), with high‐intensity ultrasonication applied to the suspension prior to casting to tune the color of the resulting films. The cholesteric CNC suspension was deposited onto a polyethylene terephthalate (PET) substrate using a laboratory‐scale blade coater and dried under ambient conditions. Finally, the PET substrate was separated, resulting in free‐standing CNC films (approximate thickness of 50 µm, Figures S1 and S2, Supporting Information) with vibrant red, green, and blue colors (Figure 1a,b and Figure S3, Supporting Information). Fabrication details are given in the Experimental Section. Their visual appearance is presented on a CIE 1931 chromaticity diagram (Figure S4 and Note S1, Supporting Information), confirming good coverage of the visible spectrum.

**Figure 2:**
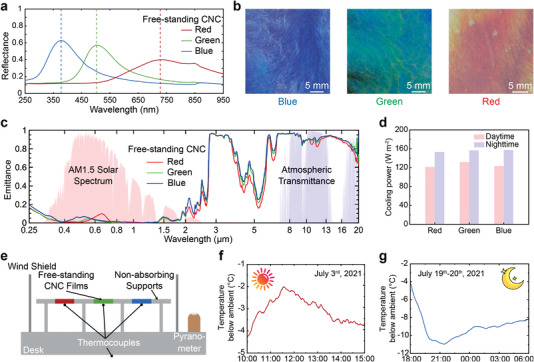
Optical and thermal characterization of the free‐standing CNC films. a) Hemispheric reflectance from 250 to 950 nm of free‐standing CNC films showing the peaks for the three representative colors. b) Macroscopic images of the blue, green, and red CNC films. c) Hemispheric emittance from 250 nm to 20 µm of the free‐standing CNC films with red, green, and blue structural color. d) Theoretical cooling power derived from samples’ optical emittance during daytime and nighttime with a solar irradiation of 900 W m^−2^. e) Schematic of the temperature measurement setup in the field test. f) Temperature below the ambient during noon time for CNC blue sample on July 3, 2021. g) Temperature below the ambient for CNC blue sample over the night on July 19–20, 2021.

To reveal the specific reflection and absorption characteristics of the CNC films across the solar spectral range, the optical responses were measured from 250 to 2000 nm by a ultraviolet (UV)–vis–NIR spectrometer (**Figure** **2**a and Figure S5, Supporting Information). The reflectance spectra of the blue, green, and red films, as measured at normal incidence, show strong peaks at 380, 500, and 740 nm, respectively (Figure 2b). Investigation of the film cross‐sections via scanning electron microscopy (SEM) revealed the characteristic Bouligand arches of the helicoidal nanostructure (Figure S1, Supporting Information), with pitch values consistent with the peak reflected wavelength, in accordance with Bragg's law. The peak reflectance of the films is saturated (i.e., approximately 50% for a left‐handed chiral nematic structure, as explained in Figure S3, Supporting Information) due to their thickness. A small amount of additional reflection was observed for the blue and green CNC films in Figure 2a, attributed to a degree of structural disorder.

CNC films have nonzero UV absorptances^[^ ^12^ ^]^ (Figure 2c), but are transparent in the vis–NIR region (Figure S5, Supporting Information), which results in an overall solar absorption of only 2.8–3.8%. The solar transmission through the CNC films can be effectively reflected by substrates, as exemplified in the next section. Their optical properties at IR wavelengths (2–20 µm) were measured using an FT–IR spectrometer, allowing assessment of any radiative heat emission (Note S2, Supporting Information). Exhibiting near‐unity hemispheric emittance in the mid‐IR range, the CNC films strongly emit at wavelengths coincident with the atmospheric transparent window (8–13 µm), enabling effective heat removal to outer space. This can be explained by the numerous functional groups of the constituent d‐glucose units within the CNCs, for example, O–H bending (*ν* = 1310–1440 cm^−1^, corresponding to *λ* = 6.9–7.6 µm), C–O stretching (1050–1310cm^−1^, 7.6–9.5 µm), and C–H bending (700–900 cm^−1^, 11.1–14.3 µm).^[^ ^13^ ^]^ Unsurprisingly, a similar emittance of 91–92%, averaged over the atmospheric transparent window, is observed for all CNC films due to their identical composition. By integrating emittance spectra and taking 900 W m^−2^ total solar irradiation at 27 °C, a heating power of 25–34 W m^−2^ is obtained. The theoretical cooling power of the three colored films can be evaluated (Figure 2d and Note S3, Supporting Information), with a daytime cooling power of 122–132 W m^−2^ and a nighttime cooling power of 153–157 W m^−2^. As such the free‐standing CNC films have significant potential as a colored radiative cooling material.

To demonstrate the radiative cooling performance in field tests, free‐standing CNC films were suspended by non‐absorbing supports made from bleached pulp sheets (Figure 2e and Figure S6, Supporting Information). The 8 × 8 cm^2^ films are large enough to avoid parasitic effects from the contact of the sample and the support. The sample temperature, ambient temperature, and solar irradiation were monitored continuously for both daytime and nighttime on the rooftop of Herrick building, Purdue University (West Lafayette, IN, USA 40.420684° N, 86.919893° W). Details of field tests are given in the Experimental Section. When exposed to a clear sky, the CNC films are cooled down without any energy input. As expected, the sub‐ambient temperatures of the CNC films are similar irrespective of their color, with an average variation of 0.62 °C among the three different colored CNC films (Figure S7, Supporting Information). On July 3, 2021, a 3.5 °C sub‐ambient temperature was recorded for the blue CNC film at midday with maximum solar irradiation of 957 W m^−2^, while a peak of 4.3 °C below ambient temperature was recorded 2 hours earlier, at 10:00 a.m. (Figure 2f and Figure S8, Supporting Information). During the night, the lowest sub‐ambient temperature observed was 11.0 °C for the blue and red CNC films (Figure 2g and Figure S9, Supporting Information). With an average sub‐ambient temperature of 3.0 °C during the day and 9.1 °C during the night, free‐standing structurally colored CNC films can be effectively employed for their radiative cooling properties.

### 1.2 Structurally Colored Cellulosic Bilayer Films for Broadband Solar Reflection

To minimize the transmission of solar radiation, the structurally colored CNC film can be deposited on a highly scattering base layer, such that any light transmitted through the CNC layer would be efficiently backscattered. Here we explore this concept by depositing the photonic CNC film on top of a porous EC film. Again, the optical properties are determined by the nanostructure of this second layer, with the high scattering efficiency arising from the presence of polydisperse pores within the EC matrix, while the repeated use of cellulose ensures low solar absorption and good IR emission.

The porous EC film is produced by an evaporation‐induced phase separation method.^[^ ^14^ ^]^ In short, by dissolving EC in a solvent (such as ethanol) and then adding a less‐volatile non‐solvent to the solution (such as water), a porous network is formed upon drying (Figure S10, Supporting Information). As such, it is possible to achieve highly scattering films by the same blade‐coating deposition method used for the CNC films (see Experimental Section). The pore sizes within the EC matrix range from hundreds of nanometers to several micrometers (Figure S11, Supporting Information), which combined with the large contrast between the refractive index of EC (*n* = 1.47)^[^ ^15^ ^]^ and the air inside the pores (*n* = 1.00), make it an effective scatterer for visible light. As such the EC film enhances light scattering across the vis–NIR spectrum, resulting in a white appearance and limited transmittance of solar radiation.

To enable the production of CNC–EC bilayer films, the surface of the EC film was activated with oxygen plasma to ensure good wetting of the aqueous CNC suspension, allowing it to be blade‐coated and dried on top of the EC film following a similar protocol to before. The distinct morphologies of each layer in this composite cellulosic film were confirmed by cross‐sectional SEM (Figure S12, Supporting Information). The strong bonding between the CNC and EC layers was validated by a shear stress test and a cross hatch adhesion test (see Experimental Section and Figures S13–S15, Supporting Information). A photograph of an EC film with a region coated with a blue CNC layer is shown in Figure 1e, revealing the distinct optical appearances of each layer. As a visual example of the cooling performance of such films, we used thermal imaging to visualize that the blue CNC–EC bilayer film has a lower surface temperature than a piece of white printer paper coated with a blue ink of the same hue (Figure 1f).

**Figure 3:**
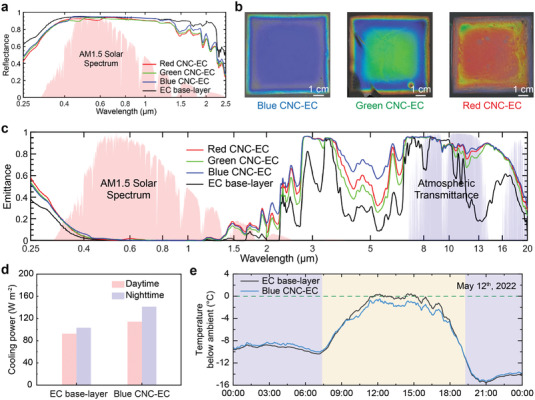
Optical and thermal characterization of CNC–EC bilayer samples. a) Hemispheric reflectance from 250 nm to 2.5 µm of an EC film and blue, green, and red CNC–EC bilayer films, showing the broadband reflection. b) Macroscopic images of the blue, green, and red CNC–EC bilayer films. Note that drying effects cause a blue shift in color at the edge of the film. c) Hemispheric emittance of the EC film and CNC–EC bilayer films from 250 nm to 20 µm. d) Theoretical cooling power derived from samples’ optical emittance spectra during daytime and nighttime with a solar irradiation of 900 W m^−2^. e) Temperature below the ambient for both the EC film and blue CNC–EC bilayer film in the field test on May 12, 2022.

In contrast to free‐standing CNC films, the red, green, and blue CNC–EC bilayer films show a broadband reflection due to the porous EC layer (**Figure** **3**a). The presence of the EC layer results in >90% solar reflection from 400 nm to 2 µm, due to backscattering from the porous structure, while maintaining near‐zero solar absorption (Figure S16, Supporting Information). Compared to the EC film alone, the addition of the CNC layer increases the absorptance in the UV by just 3.5–4.9%, while in the mid‐IR region there is a large rise in emittance of 16.6–20.5% (Figure S16, Supporting Information). Note that the small variation in performance for different colored films can be attributed to differing film thicknesses (Figure S2, Supporting Information). The theoretical cooling powers of the EC film and the CNC–EC bilayer film were evaluated using the same analytical model as before (Note S3, Supporting Information). An overall higher cooling power is obtained for the CNC–EC bilayer (Figure 3d), suggesting that the improved IR emittance overwhelms the small increase in UV absorption (Figure 3c). This trend in the theoretical cooling powers is also reflected in a comparative field test, where the temperatures of an EC film and a blue CNC–EC bilayer film are compared to the ambient temperature over a 24 h period (May 12^th^, 2022, see Figure 3e). We measured that the EC film had a temperature close to ambient (≈32 °C) from 1 to 3 p.m., while over the same time period the blue CNC–EC bilayer film had an average sub‐ambient temperature of 1.4 °C (Figure 3e and Figure S17, Supporting Information). Furthermore, while both films were effective at radiating heat during the night, the blue CNC–EC bilayer film displayed the lowest temperature, measured as 15.4 °C below ambient. As such, the sub‐ambient radiative cooling achieved by CNC–EC bilayer films throughout the diurnal cycle strongly validates the use of such sustainable materials for real‐world passive cooling applications.

Interestingly, casting a structurally colored CNC film on top of the white EC base layer gives rise to a more complex angle‐dependent optical response than that seen for free‐standing CNC films, as exemplified when rotating a red CNC–EC bilayer film under fixed illumination (Supplementary Video 3). We quantified the optical response of the CNC–EC films using angle‐dependent optical spectroscopy (see Experimental Section and Figures S18–S22, Supporting Information). At specular angles (Figure S19, Supporting Information), the CNC–EC bilayer films exhibit the typical structural color observed for free‐standing CNC films (with a blue‐shift at higher angles due to Fergason's law). Notably, at off‐specular angles (Figure S20, Supporting Information) the corresponding negative color for each CNC film is observed: in the case of a CNC–EC film that is blue under normal illumination, the same film appears yellowish (i.e., white minus blue) at most other angles of observation (as illustrated in Figure S21, Supporting Information). The angle‐resolved spectroscopy also reveals a rich interplay of optical effects between the backscattering EC layer and the CNC cholesteric structure, as illustrated in Figure S22, Supporting Information.

### 1.3 Large‐Scale Production of Cellulosic Bilayer Coatings by Roll‐to‐Roll Deposition

**Figure 4:**
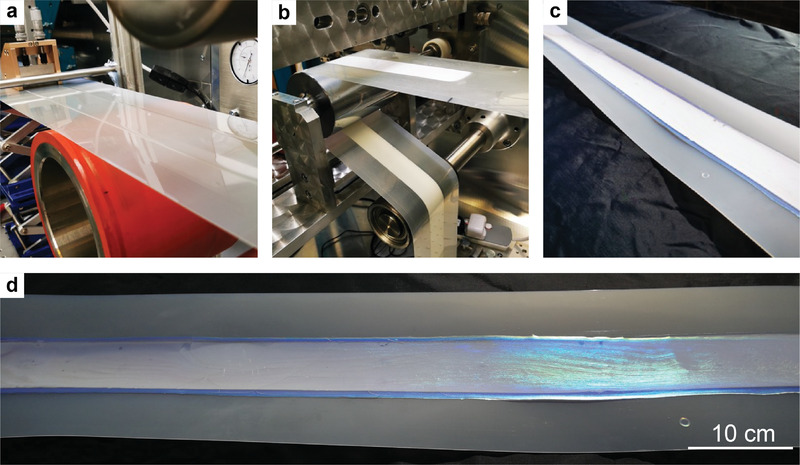
Large‐scale R2R fabrication of structurally colored cellulosic films. a) Blade casting of the EC solution on the PET web (i.e., substrate). b) Rewinding of the dry EC film attached on the PET web. c) Large‐scale CNC–EC bilayer film. Due to the off‐specular lighting, the film appears yellow for the most part except at the edges where the blue structural color from the CNC layer is visible. d) Same sample as in (c), observed under natural light to reveal the vibrant blue coloration when at specular reflection.

Sub‐ambient cooling throughout the day and night for both free‐standing CNC films and CNC–EC bilayer films promotes their use in multiple applications, such as decorative cladding for buildings or in automotive paint. To demonstrate the scalability of the structurally colored bilayer cellulosic films, large‐scale prototypes were fabricated using a R2R deposition method. The process is summarized here, with technical parameters provided in the Experimental Section. First, the EC solution was deposited over a PET substrate by a standard blade‐casting method mounted on a R2R machine (**Figure** **4**a). The solution was left to dry at ambient temperature for around 2 h to yield a white EC film. After complete drying, the EC‐coated PET substrate coated was rewound (Figure 4b) and corona‐treated to activate the surface. Finally, a CNC suspension was deposited on top of this EC layer using a previously reported method.^[^ ^11^ ^]^ After complete drying of the CNC suspension at ambient temperature, a R2R‐cast CNC–EC bilayer film with a length greater than two meters was obtained (Figure 4c). Consistent with the laboratory‐scale samples cast from the same formulation, the R2R‐cast CNC–EC bilayer film displayed uniform structural coloration under solar illumination, with a vibrant blue reflection at specular angles and a light‐yellow appearance at off‐specular angles (Figure 4d). The optical performance, in terms of visible reflection, solar absorption, and IR emittance across the atmospheric transparent window, was found to be consistent between the two deposition methods (Figures S23 and S24, Supporting Information). With the promising structural coloration, radiative cooling performance, and large‐scale manufacture, the cellulosic films can be widely applied to resolve the dilemma between coloration and thermal management in architecture and automotive design.

## 2 Conclusion

In this study, we have demonstrated that the trade‐off between vivid coloration and sub‐ambient DRC can be overcome by exploiting structural color. This was accomplished by combining the inherent mid‐IR emission and negligible absorption of CNCs, with their ability to self‐assemble into a cholesteric nanostructure. Furthermore, we removed solar transmission by casting the CNC film on top of a scattering base layer composed of EC. The optical properties of such CNC–EC bilayer films were elaborated in terms of the structural coloration of the CNC layer, the diffusive broadband scattering of the EC layer, and the close‐to‐unity emittance in the atmospheric transparent window inherent to cellulose, allowing for the quantification of the cooling power. By fabricating the CNC–EC bilayer film using a R2R approach, we validated that this sustainable photonic material can be commercially exploited at large‐scale for colored DRC. Overall, the scalable, cost‐effective, and sustainable origin of this all‐cellulose composite makes it well placed for sub‐ambient radiative cooling, offering a way to mitigate global warming and achieve carbon neutrality.

## 3 Experimental Section

### Preparation of Cellulose Nanocrystal Suspension

The CNC suspension was purchased from the University of Maine Process Development Center and processed according to a previously reported method.^[^ ^11^ ^]^ In brief, the suspension was diluted with ultrapure water to 6 wt%, followed by tip sonication in an ice bath using an ultrasonic disintegrator (Fisherbrand 505 Sonic Dismembrator, 500 W, amplitude = 40%, tip diameter = 12.7 mm). The duration of sonication was used to tune the CNC properties, allowing for suspensions to be formulated that produce blue, green, and red photonic films after complete drying. These suspensions were left to phase separate for several days, after which the anisotropic phase was collected and used for film casting.

### Preparation of Ethylcellulose Solution

EC powder (Sigma‐Aldrich 247499), ethanol, and water were mixed with a mass ratio of 1:5:1, and dissolved by bath sonication to form a clear solution.

### Laboratory‐Scale Blade Casting

Laboratory‐scale blade‐cast CNC films were prepared using a bespoke blade‐coater with a maximum casting length of approximately 30 cm. This coater is composed of a motor (Reliance Cool Motion Stage) that can move a flat stage along a track, above which a coating applicator (BEVS 1806/A50) was mounted at a fixed position. The CNC suspension was deposited through a coating gap of 1100 µm over a PET substrate (HIFI Film PMX727), the surface of which was pre‐activated to control wetting using a plasma etcher (EMITECH K1050X, vacuumed air atmosphere, 50 W, 5 min). After complete drying, the CNC film was peeled off the PET substrate. For EC and CNC–EC films, a layer of the prepared EC solution was blade‐coated onto a glass slide with a 700 µm coating gap. After drying in the ambient environment, a porous EC film was formed that could be either detached (giving a white EC film) or further coated with a layer of CNC suspension to yield a colored CNC–EC bilayer film. In the latter case, the surface of the EC film was plasma‐treated before the deposition of the CNC suspension to introduce more polar groups (e.g., –OH), which both improves wetting of the CNC suspension and strengthens the hydrogen bonding between the EC and CNC layers in the resultant composite film. In the case of the blue and green films, a 700 µm coating gap was used while a 900 µm gap was used to prepare the red film.

### Large‐Scale R2R Fabrication of CNC–EC Bilayer Film

Large‐scale fabrication of CNC–EC bilayer film was achieved using a modified R2R casting system (Coatema Coating Machinery, Smartcoater 28). A blade (BEVS 1806A/100) fixed across the R2R pathway was used for the deposition of the EC layer over the PET web while a custom‐made slot‐die (casting width = 10 cm, internal reservoir = 22 mL) allowed for the deposition of the CNC layer over the EC film. The slot‐die was made of two screw‐joined aluminum plates separated by a 125 µm‐thick spacer shim configured with a slot opening of 100 mm and positioned perpendicular to the web. Web‐holders were placed so that the average distance between each support was 30 cm. The substrate was leveled before casting using a bullseye bubble level (Thorlabs LVL01) at several positions along the web path and at the middle of the width. The web was translated in order to deposit the EC solution over a distance of approximately 2 m before stopping the translation of the web, and allowing the material to dry under ambient conditions. After complete drying and formation of the porous structure, the web and attached EC film were rewound and corona‐treated before depositing the CNC suspension through a slot‐die. The CNC solution was similarly dried under ambient conditions to yield a photonic CNC–EC bilayer film.

### Polarized Optical Microscopy

Polarized optical microscopy (Zeiss Axio Scope A1) images of the CNC films were taken using a 20× objective (Zeiss EC Epiplan APOCHROMAT, NA = 0.3). The light reflected by the CNC and CNC–EC films passed through a quarter‐wave plate and an orientable linearly polarizing filter, which can together filter either the left‐ or the right‐circularly polarized light reflected by the sample (denoted LCP and RCP, respectively). A beamsplitter allowed the light to be directed at a CMOS camera (UI‐3580LE, IDS) and a fiber‐coupled spectrometer (Avantes AvaSpec HS2048), which collects light from a defined region of the microscope field of view. A 600 µm core optical fiber (Thorlabs FC‐UV600‐2‐SR) was used for measuring the reflectance from the films via a magnifying lens (Thorlabs AC254‐050‐A). As a result, spectra were acquired with a spot size of ≈100 µm. The spectra were normalized to the reflection from a silver mirror (Thorlabs PF10‐03‐P01) in one polarization channel (LCP), such that a perfectly aligned cholesteric sample would reflect 100% LCP intensity.

### Optical Spectrum Characterization

The total hemispheric transmittance and reflectance from 0.25 to 2 µm were measured by Perkin Elmer Lambda 950 UV–vis–NIR spectrometer (±0.02% uncertainty) with an integrating sphere, using a certified Spectralon diffuse reflectance standard. The total hemispheric transmittance and reflectance between 2 and 20 µm were measured with ± 0.5% uncertainty by a FT–IR spectrometer (Nicolet iS50 FTIR) with an integrating sphere (PIKE Technologies). Measurements were triplicated at different spots and averaged.

### Goniometer Measurement

The angle‐resolved optical response of the films was measured by optical spectroscopy on a custom goniometer setup. A broadband xenon lamp (HPX2000, Ocean Optics) was coupled to a reflective collimator (RC08SMA‐F01, Thorlabs) via a 100 µm optic fiber (FC‐UV100‐2, Avantes) and used to illuminate the sample with a spot diameter *Ø* ≈1 mm. The reflected light was collected using another collimator coupled to a UV–vis spectrometer (AvaSpec‐HS2048, Avantes) via a 1000 µm optic fiber (FC‐UV1000‐2, Avantes). The recorded light intensity was normalized to a white Lambertian diffuser and the exposure time was adjusted automatically using a high dynamic range method. Three goniometer measurement modes were used, as illustrated in Figure S18, Supporting Information. For specular measurements, the angles of illumination and collection (relative to the sample axis) were increased symmetrically about the sample axis, producing the scans shown in Figure S19, Supporting Information. For off‐specular measurements, the sample was illuminated at a fixed angle of 30° to the sample axis while the collection angle was varied, producing the scans shown in Figure S20, Supporting Information. For Supplementary Videos 1‐3, the respective blue, green and red bilayer films were rotated while the illumination and collection angles were kept fixed.

### Field Test Experiments

The field tests were conducted on the rooftop of the Herrick Lab at Purdue University (40.420684° N, 86.919893° W) between July 2021 and May 2022. The free‐standing CNC films were suspended to avoid parasite heating from the environment. The supporting frame was made of bleached pulp sheets (≈1 mm thick) with an effective solar reflectance of 0.931. Free‐standing CNC films with red, green, and blue colors were cut to 8 × 8 cm^2^ and suspended on top of the 7 × 7 cm^2^ aperture. A gauge 40 T‐type thermocouple is attached to the back of each sample. A tiny square of the bleached pulp with approximately 5 × 5× 0.25 mm^3^ was inserted between the sample and the thermocouple to prevent the direct solar heating of the thermocouple tip without significantly affecting the temperature measurement. The thermal resistance was estimated as 0.0025 m^2^ K W^−1^ assuming the thermal conductivity of the bleached pulp was 0.10 W m^−1^ K^−1^.^[^ ^16^ ^]^ A temperature difference of 0.25 °C between the CNC films and thermocouples could be estimated with a cooling power of 100 W m^−2^, which means the bleached pulp slices do not significantly affect the temperature measurement. A windshield made of polyethylene foil (approximately 10 µm thick) surrounded the suspended samples to reduce the convective heat loss. When testing the EC film and CNC–EC bilayer films, each sample of 8 × 8 cm^2^ was placed in an insulated Styrofoam box with reflective covers to avoid parasite heating (Figure 3f). A polyethylene film of approximately 10 µm thick was used to seal the sample compartment to minimize the convection loss without interfering with the radiative heat exchange. The backside temperatures of the EC base layer and CNC–EC bilayer films are monitored by the T‐type thermocouples and recorded by a data acquisition unit (Keysight DAQ973A). The local ambient temperature, relative humidity, wind speed, and solar irradiance are monitored by a weather station with an integrated Stevenson screen (HD52.3DP17R). The sub‐ambient temperature was derived from the difference between the sample temperature and the ambient temperature. The sub‐ambient temperatures were smoothed by moving average of 50 and 10 data points for field tests of the free‐standing CNC and CNC–EC bilayer films, respectively.

### Shear Stress Test

A blue CNC–EC bilayer film coated on a copper sheet (thickness 0.32 mm) was prepared. To measure the shear stress between the CNC and EC layers, a tensile meter (MTS Criterion Series 40 Electromechanical Universal Test Systems) stretched the two layers in the shear direction. For fixtures, the EC side of the CNC–EC composite had a copper sheet while the CNC side was adhered to a steel sheet by double‐sided tape (Figure S14, Supporting Information). The applied shear load and displacement of the crosshead were recorded. The tests were repeated three times on three different specimens. Since the failure occurred at adhesion in the CNC–steel interface, the shear stress was calculated based on the area of adhesion (446.2–519.2 mm^2^) between the CNC layer and steel sheet. The results in Figure S15, Supporting Information, indicated a strong bond between the CNC and EC layer, estimated to exceed 176 kPa.

### Cross Hatch Adhesion Test

The cross hatch adhesion test was carried out following the ISO2409‐1992 standard, using a commercial cross hatch tester (CGOLDENWALL multi‐blade cutter, with spacing 1.00 ± 0.01 mm, addendum straightness 0.003 mm, tooth tip width 0.05 mm). First, the sample was placed on a tablet of sufficient hardness. Second, the surface was cut by applying uniform force while keeping the multi‐blade cutter perpendicular to the sample surface. The cut was repeated after a 90° sample rotation to form a grid pattern. Then, a soft brush was used to gently brush the two diagonal lines of the grid backward and forward five times. Afterward, a 19 mm wide and transparent adhesion tape (600 from 3M) was put over the entire grid and torn off at the minimum angle. The tests were repeated three times for bilayer on copper samples and free‐standing bilayer samples. According to the adhesive peeling area ratio, the samples all showed less than 5% damage, indicating strong bonding between the CNC and EC layers (Figure S13, Supporting Information).

### Mechanical Strength Measurement

The mechanical tensile strengths of blue CNC–EC bilayer films and EC base‐layer samples were measured by a tensile meter (MTS Criterion Series 40 Electromechanical Universal Test Systems). Three slices (1 × 5 cm^2^) of the CNC–EC bilayer and the EC base layer were prepared from the same sample. The thickness and width of each slice were recorded before testing by a caliper. During the test, the applied load and the sample elongation were monitored simultaneously. The strain was calculated by the displacement over the original length of the test section of the sample slice. The tensile stress was calculated by the load over the cross‐sectional area of each sample slice. The mechanical strength was taken as the maximum value on the strain–stress curve (ultimate strength). The strain–stress curves of the two samples are shown in Figure S25, Supporting Information. The average value and the uncertainties of the blue CNC–EC bilayer film and EC base layer were 18.40 ± 1.58 and 10.98 ± 0.19 MPa, respectively.

### Morphology and Thickness Determination

Film cross‐sections were achieved by bending the film after rapid freezing in cryogenic liquid nitrogen. The microscale and nanoscale morphological features were revealed by SEM, using an FEI Nova NanoSEM and Quanta 3D FEG (for Figure 1 and Figure S11 and S12, Supporting Information) and using a Tescan MIRA3 FEG‐SEM (for Figure S1, Supporting Information). Film thicknesses were extracted from the images using ImageJ software.

### Statistical Analysis

For optical responses, the UV–vis‐NIR and FT–IR measurements were repeated three times for each sample at three different locations and the mean values were reported in Figure 2b. The variation of the three optical measurements for each sample was less than 0.1%. For results from polarized optical microscopy, each reported spectrum was the average of at least ten measurements. For the R2R‐cast film, the reported spectrum was the average of at least ten measurements on every four different locations along the R2R sample. The data collected in the field test were smoothed by moving average of 50 data points for free‐standing CNC films (samples in free‐standing test setup were more susceptible to the change of ambient temperature and wind speed) and 10 data points for CNC–EC bilayer films. The raw temperature data were reported in Figures S7, S9, and S17, Supporting Information. The mechanical tensile tests were repeated with three specimens from each sample. The error value of each sample was taken as half of the range of its result dataset.

## Conflict of Interest

The authors declare no conflict of interest.

## Supporting information

Supporting Information

Supporting Information

Supplemental Video 1

Supplemental Video 2

Supplemental Video 3

## Data Availability

The data supporting the findings of this study are openly available from the University of Cambridge data repository (https://doi.org/10.17863/CAM.85818).
