# Supplementary material for: Structurally Colored Radiative Cooling Cellulosic Films
Source: Adv Sci (Weinh). 2022 Jul 17;9(26):2202061. doi: 10.1002/advs.202202061 (PMC9475522; doi:10.1002/advs.202202061)
Supplement: Supplementary file 1 — Supporting Information [file ADVS-9-2202061-s004.pdf]

Supplementary Materials

Table of contents:

Part I: Supplementary Discussions
Note S1. Free-standing CNC chromaticity
Note S2. Optical characterization
Note S3. Theoretical cooling power estimation

Part II: Supplementary Figures
Fig. S1. Cross-section SEM images of CNC films (*top*) and CNC-EC bilayer films (*bottom*). These images were used for the thickness measurement reported in Fig. S2.
Fig. S2. Bar chart summarizing the thickness of films reported in the article. The colored portion refers to the CNC film, while grey bars correspond to the EC layer.
Fig. S3. Polarized optical microscope images and corresponding micro-spectra, recorded through left- and right- circularity polarized filters (denoted LCP and RCP), for (a) the CNC films and (b) the CNC-EC bilayer films and EC film alone.
Fig. S4. CIE 1931 chromaticity diagram of the free-standing red, green and blue CNC films.
Fig. S5. (a) Reflectance and (b) transmittance of red, green, and blue free-standing CNC films in the UV-VIS-IR spectral range.
Fig. S6. (a) Top view of the field test setup measuring the suspended CNC films. (b) Top view of temperature measurement system for the EC base-layer and CNC-EC bilayer films. (c) Photo of the weather station used in the field test.
Fig. S7. Daytime (a) raw temperature data and (b) temperature below the ambient of the blue, green, and red free-standing CNC films, during the field test on July 3rd, 2021.
Fig. S8. Solar irradiation during the daytime field test on July 3rd, 2021.
Fig. S9. Nighttime (a) raw temperature data and (b) temperature below the ambient of blue and red free-standing CNC films, during the field test on July 19th – 20th, 2021.
Fig. S10. Schematic of the formation of the porous ethylcellulose film.
Fig. S11. Cross-sectional SEM image of the EC film showing (a) pore sizes from hundreds of nanometers to several microns and, (b) an enhanced view of the pores at higher magnification.
Fig. S12. Cross-sectional SEM image of the CNC-EC bilayer film, showing the clearly defined interface between the two nanostructures (top: CNC, bottom: EC).
Fig. S13. Results of cross hatch adhesion test of (a) blue CNC-EC on a copper sheet with peeled tape on its left and (b) a free-standing blue CNC-EC bilayer films with peeled tape on its right. The structurally colored parts are the samples that underwent the cross hatch test.
Fig. S14. Schematic of shear stress test for blue CNC-EC on a copper sheet.
Fig. S15. Shear stress vs. displacement for blue CNC-EC on a copper sheet.
Fig. S16. (a) Emittance, (b) reflectance, and (c) transmittance of the EC film and CNC-EC bilayer films in the UV-VIS-IR spectral range.

Fig. S17. (a) Raw temperature data and (b) solar irradiation during the 24-hour field test on May 12th, 2022.

Fig. S18. Schematic of the illumination and collection procedures for angle-resolved spectroscopy (also referred to as goniometry), shown relative to a fixed frame of reference for the film.

Fig. S19. Specular scans of (a) red, (b) green, and (c) blue CNC-EC bilayer films.

Fig. S20. Off-specular scans of (a) an EC film, and (b) red, (c) green, (d) blue CNC-EC bilayer films with the illumination angle fixed at 30° relative to the film normal.

Fig. S21. Photograph of the blue CNC-EC bilayer film showing a yellowish color at off-specular angles.

Fig. S22. Schematic illustrating various contributions to the angle-dependent optical response of EC-CNC films.

Fig. S23. Polarized optical microscope images and corresponding micro-spectra, recorded through left- and right- circularity polarized filters (denoted LCP and RCP), for the laboratory-scale blade-cast CNC film and the R2R-cast CNC-EC bilayer film.

Fig. S24. Hemispheric emittance of the blue CNC-EC bilayer films prepared on laboratory-scale and large-scale from 250 nm to 20 µm.

Fig. S25. (a) Averaged tensile strength and (b) strain-stress curves of blue CNC-EC bilayer films and EC base-layer.

Supplementary Discussions

Note S1. Free-standing CNC chromaticity

To confirm the color response to human eyes, the reflectance spectra in the visible range (360-830 nm) of free-standing CNC films are converted to tristimulus value *X*, *Y*, and *Z* by the CIE 1964 color-matching functions ($\bar{x}$, $\bar{y}$, and $\bar{z}$ in Eq. S1-3).

$$X = 100 \frac{\int I(\lambda)\rho(\lambda)\bar{x}(\lambda)d\lambda}{\int I(\lambda)\bar{y}(\lambda)d\lambda} \tag{S1}$$

$$Y = 100 \frac{\int I(\lambda)\rho(\lambda)\bar{y}(\lambda)d\lambda}{\int I(\lambda)\bar{y}(\lambda)d\lambda} \tag{S2}$$

$$Z = 100 \frac{\int I(\lambda)\rho(\lambda)\bar{z}(\lambda)d\lambda}{\int I(\lambda)\bar{y}(\lambda)d\lambda} \tag{S3}$$

$I(\lambda)$ represents the illumination source spectrum and the solar spectrum is used as a natural light condition. The colors can be located in CIE 1946 color space with normalized chromaticity parameters *x* and *y* as:

$$x = \frac{X}{X+Y+Z} \tag{S4}$$

$$y = \frac{Y}{X+Y+Z} \tag{S5}$$

Note S2. Optical characterization

The emittance spectrum can be derived from the transmittance and reflectance spectra by the thermal equilibrium principle (Eq. S6) and Kirchhoff's Law, where emittance is equivalent to absorptance.

$$\rho_{eff} = \frac{\int_0^\infty \rho(\lambda) I_{solar} \mathrm{d}\lambda}{\int_0^\infty I_{solar} \mathrm{d}\lambda}, \tau_{eff} = \frac{\int_0^\infty \tau(\lambda) I_{solar} \mathrm{d}\lambda}{\int_0^\infty I_{solar} \mathrm{d}\lambda}, \alpha_{eff} = \frac{\int_0^\infty \alpha(\lambda) I_{solar} \mathrm{d}\lambda}{\int_0^\infty I_{solar} \mathrm{d}\lambda}, \alpha_{eff} + \rho_{eff} + \tau_{eff} = 1 \tag{S6}$$

where α, ρ, and τ are absorptance, reflectance, and transmittance respectively.

Note S3. Theoretical cooling power estimation

The net cooling power ($P_{cooling}$) of the sample can be estimated from

$$P_{cooling} = P_{rad} - P_{sun} - P_{atm} - P_{conv} \tag{S7}$$

where,

P_{rad}: the power density of thermal radiation emitted by the cellulose sample,

P_{sun}: the heating power density from solar irradiation,

P_{atm}: the power density of downward thermal radiation from the atmosphere,

P_{conv}: the effective power density loss including convection and conduction from the cellulose sample,

P_{rad} can be derived from the measured emittance spectrum of the sample by

$$P_{rad}(T) = \int_0^\infty \varepsilon_s(\lambda) I_{\mathrm{BB}}(T,\lambda)\mathrm{d}\lambda \quad \text{(S8)}$$

where $\varepsilon_s(\lambda)$ is the sample emittance and I_{BB} is blackbody radiation intensity as a function of emitter temperature according to Planck's law. P_{sun} from solar irradiation is calculated by integrating sample emittance over the Air Mass 1.5 (AM1.5) solar spectrum as,

$$P_{sun}(T) = \int_0^\infty \varepsilon_s(\lambda) I_{sun}(\lambda)\mathrm{d}\lambda \quad \text{(S9)}$$

P_{atm} is the amount of power emitted from the atmosphere and absorbed by the sample. The emittance spectra of the atmosphere and the cellulose sample are used (Eq. S10).

$$P_{atm}(T_{atm}) = \int_0^{\pi/2}\int_0^\infty 2\pi\sin(\theta)\cos(\theta)\varepsilon_s(\lambda,\theta)\varepsilon_{atm}(\lambda,\theta)I_{\mathrm{BB}}(T_{atm},\lambda)\mathrm{d}\lambda\mathrm{d}\theta \quad \text{(S10)}$$

where the angular part of the atmospheric emittance can be obtained by $\varepsilon_{atm}(\lambda,\theta) = 1-\tau(\theta)^{1/\cos\theta}$ where $\tau(\theta)$ is the angular atmospheric transmittance.[1]

The P_{conv} can be evaluated via the sample temperature, ambient temperature, and the effective heat transfer coefficient h as

$$P_{conv} = h(T\text{-}T_{amb}) \quad \text{(S11)}$$

Here, we evaluate and compare the isothermal theoretical cooling power of the samples assuming the same temperature for the sample and the ambient such that the convection loss term can be neglected.

Supplementary Figures

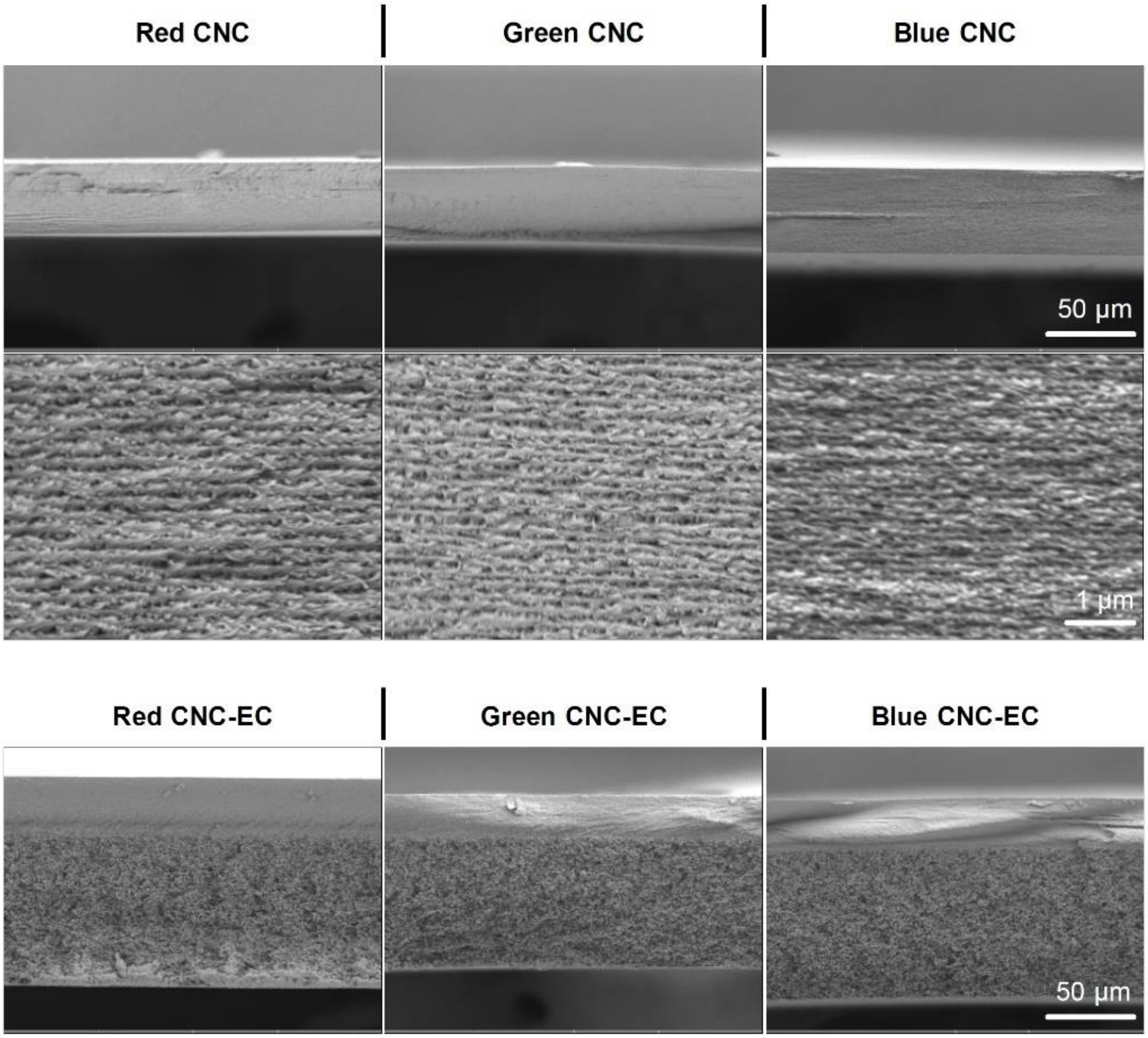

Fig. S1. Cross-section SEM images of CNC films (*top*) and CNC-EC bilayer films (*bottom*). These images were used for the thickness measurement reported in Fig. S2.

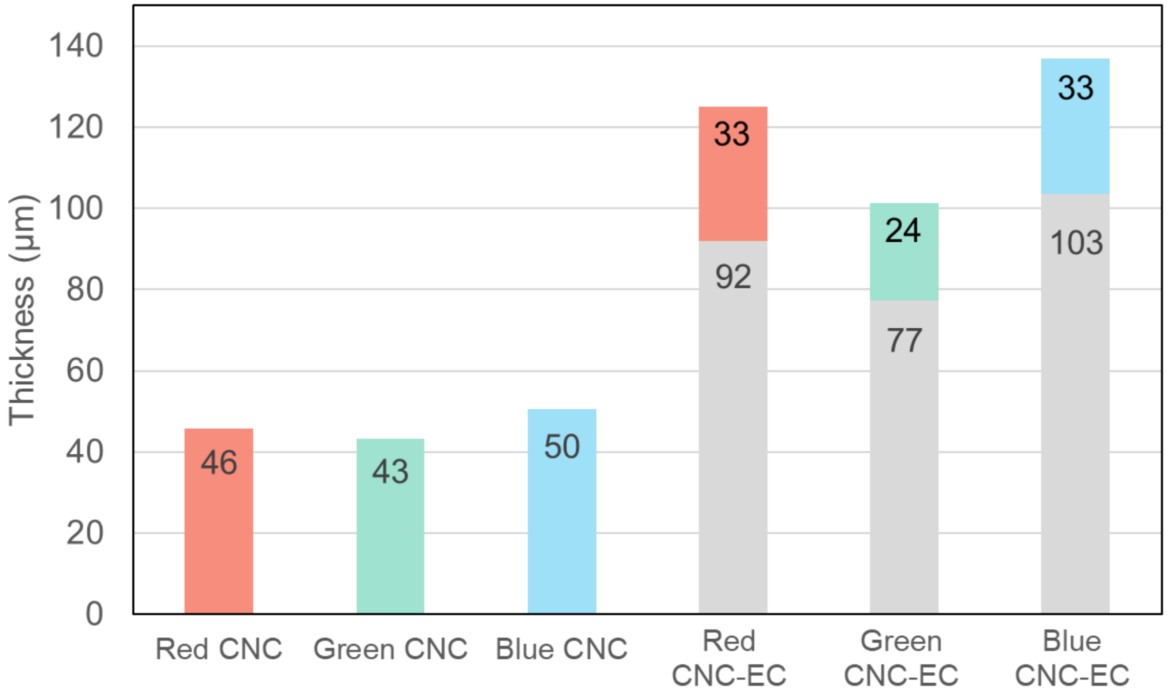

Fig. S2. Bar chart summarizing the thickness of films reported in the article. The colored portion refers to the CNC film, while grey bars correspond to the EC layer.

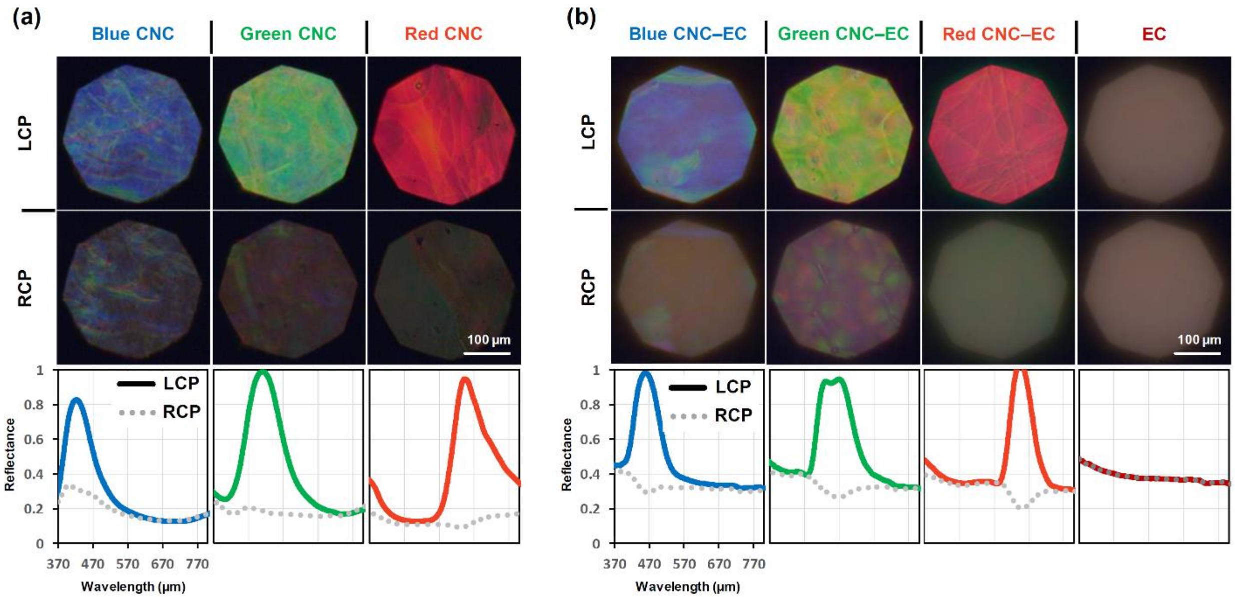

Fig. S3. Polarized optical microscope images and corresponding micro-spectra, recorded through left- and right- circularity polarized filters (denoted LCP and RCP), for **(a)** the CNC films and **(b)** the CNC-EC bilayer films and EC film alone. Each reported spectrum is the average of at least 10 measurements. Note that an ideal cholesteric CNC film contains a well-aligned left-handed helicoidal structure and as such would reflect 100% of LCP light and 0% of RCP light. As such the theoretical maximum measured reflectivity at normal incidence is 50% of unpolarized light. Note the baseline arises from the specular reflection of the air-film interface.

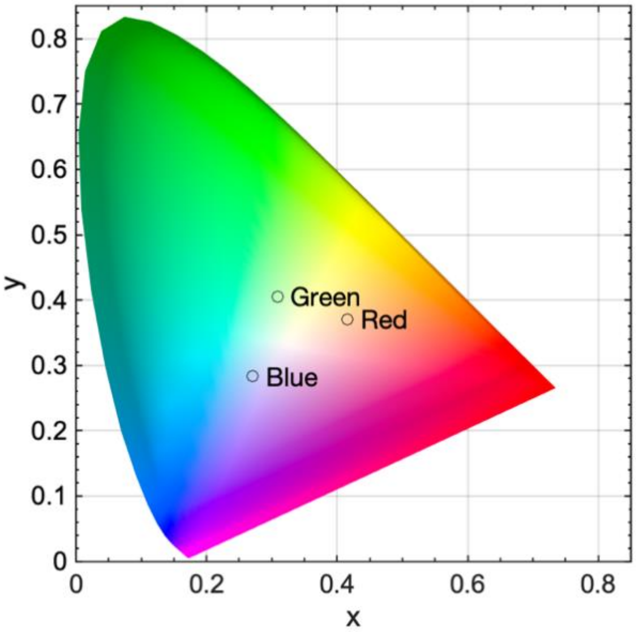

Fig. S4. CIE 1931 chromaticity diagram of the free-standing red, green and blue CNC films.

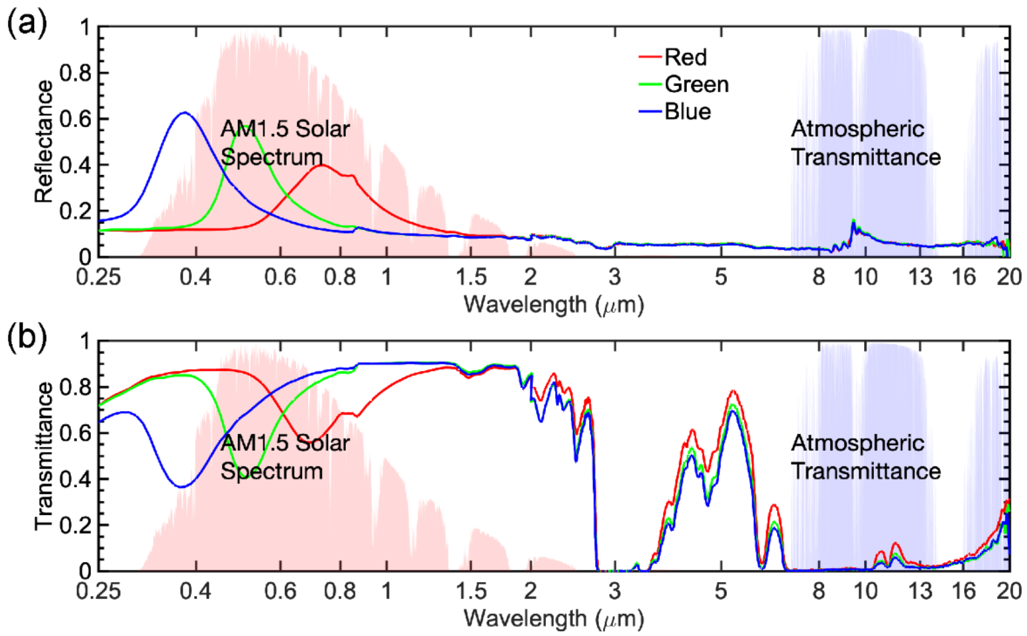

Fig. S5. (a) Reflectance and **(b)** transmittance of red, green, and blue free-standing CNC films in the UV-VIS-IR spectral range.

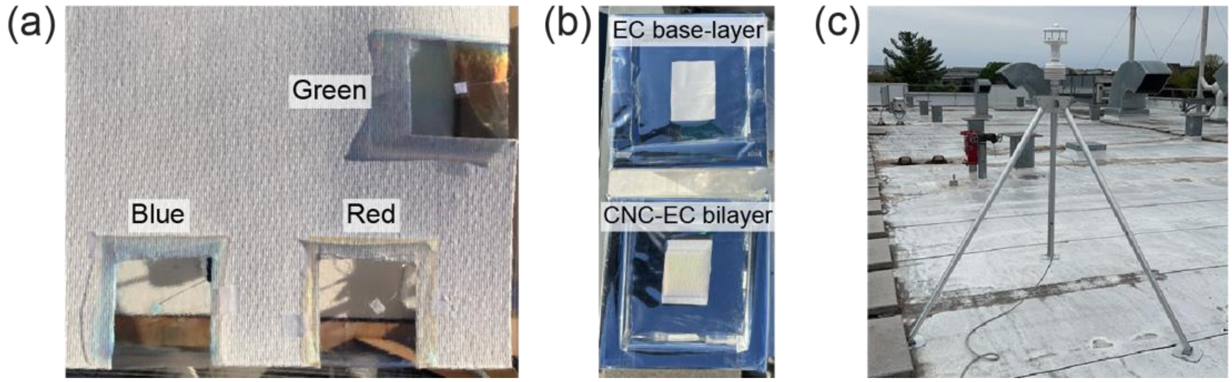

Fig. S6. (a) Top view of the field test setup measuring the suspended CNC films. **(b)** Top view of temperature measurement system for the EC base-layer and CNC-EC bilayer films. **(c)** Photo of the weather station used in the field test.

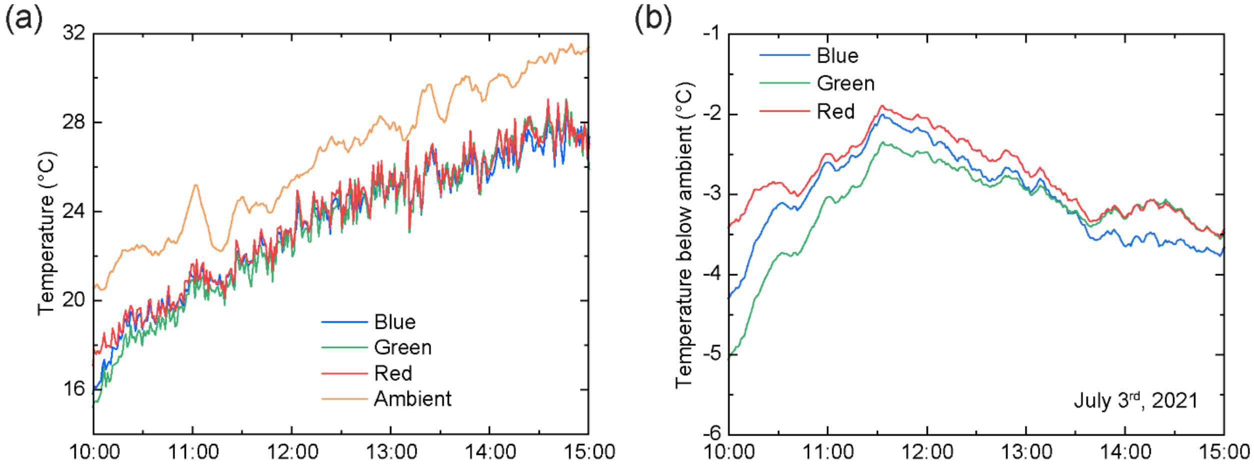

Fig. S7. Daytime **(a)** raw temperature data and **(b)** temperature below the ambient of the blue, green, and red free-standing CNC films, during the field test on July 3rd, 2021.

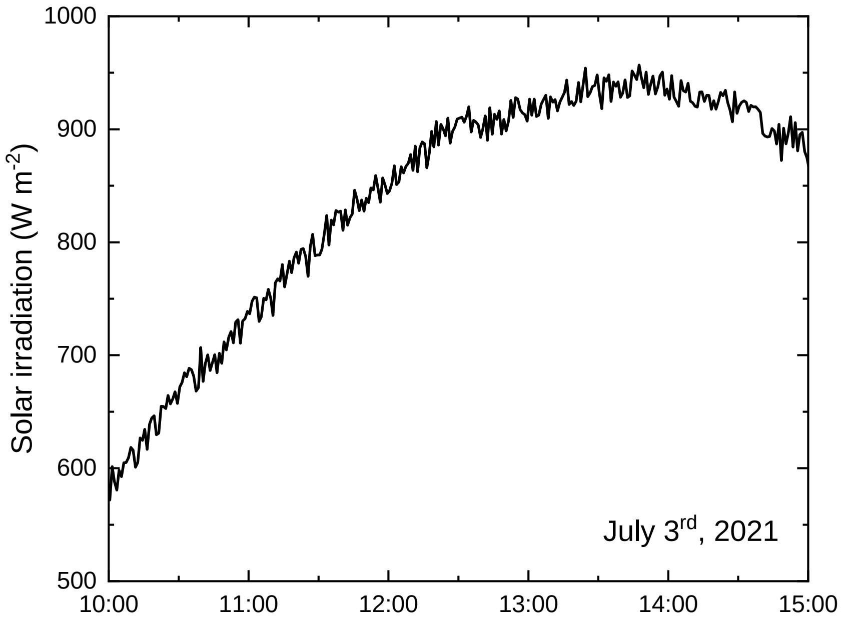

Fig. S8. Solar irradiation during the daytime field test on July 3rd, 2021.

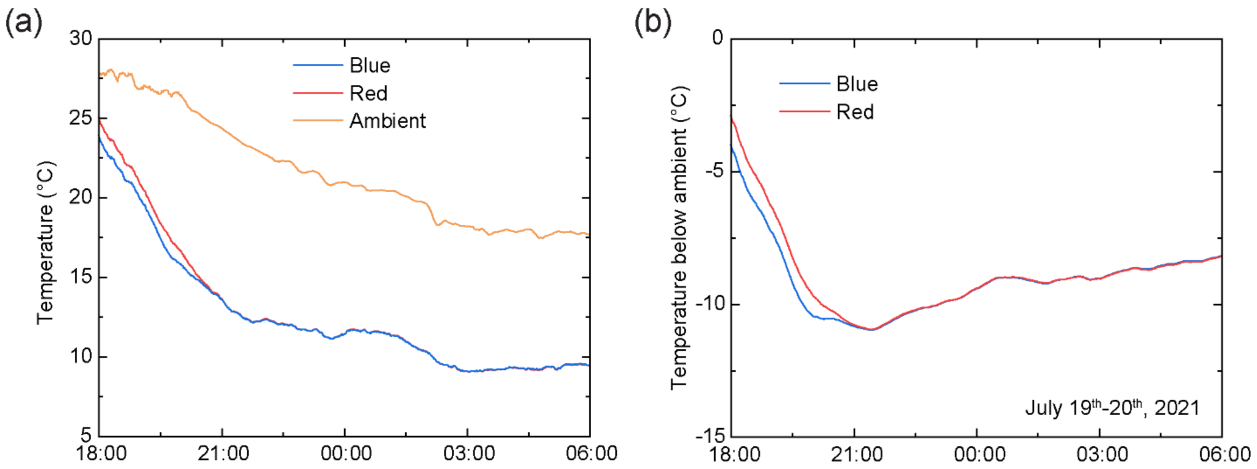

Fig. S9. Nighttime **(a)** raw temperature data and **(b)** temperature below the ambient of blue and red free-standing CNC films, during the field test on July 19th – 20th, 2021.

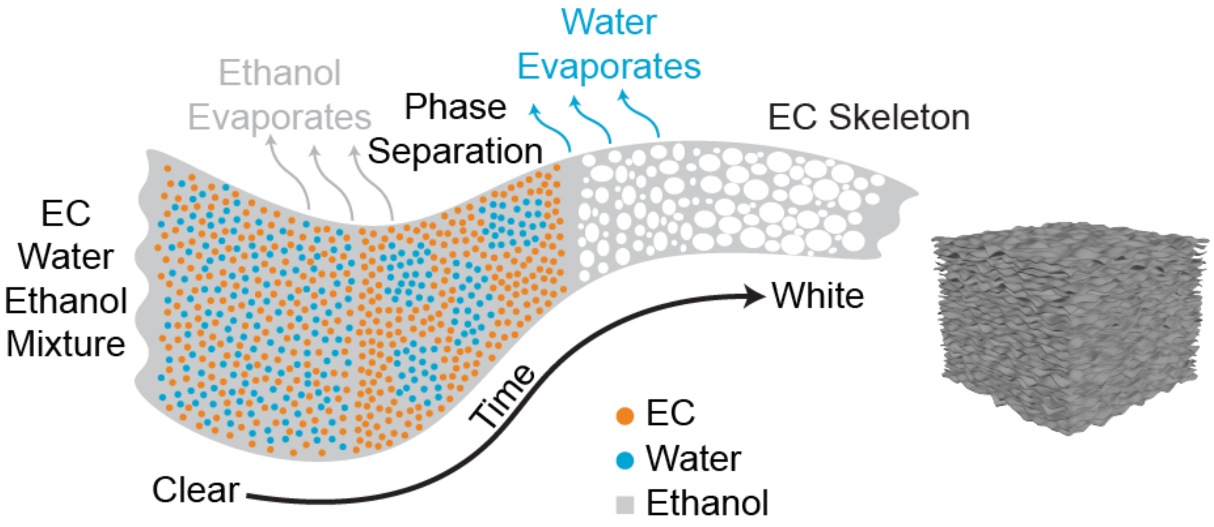

Fig. S10. Schematic of the formation of the porous ethylcellulose film.

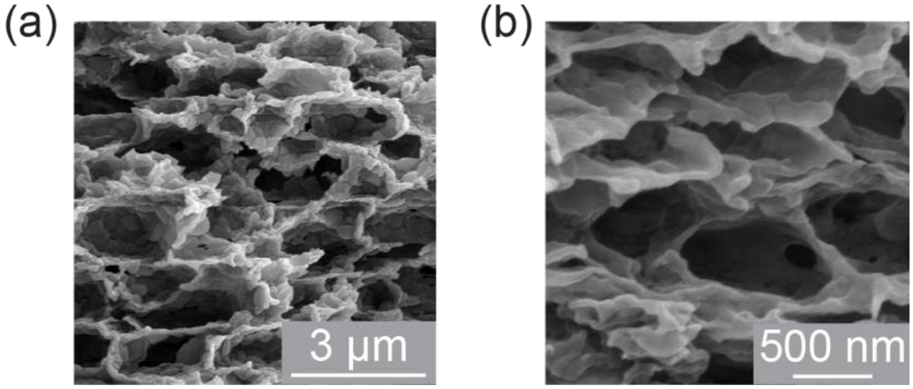

Fig. S11. Cross-sectional SEM image of the EC film showing **(a)** pore sizes from hundreds of nanometers to several microns and, **(b)** an enhanced view of the pores at higher magnification.

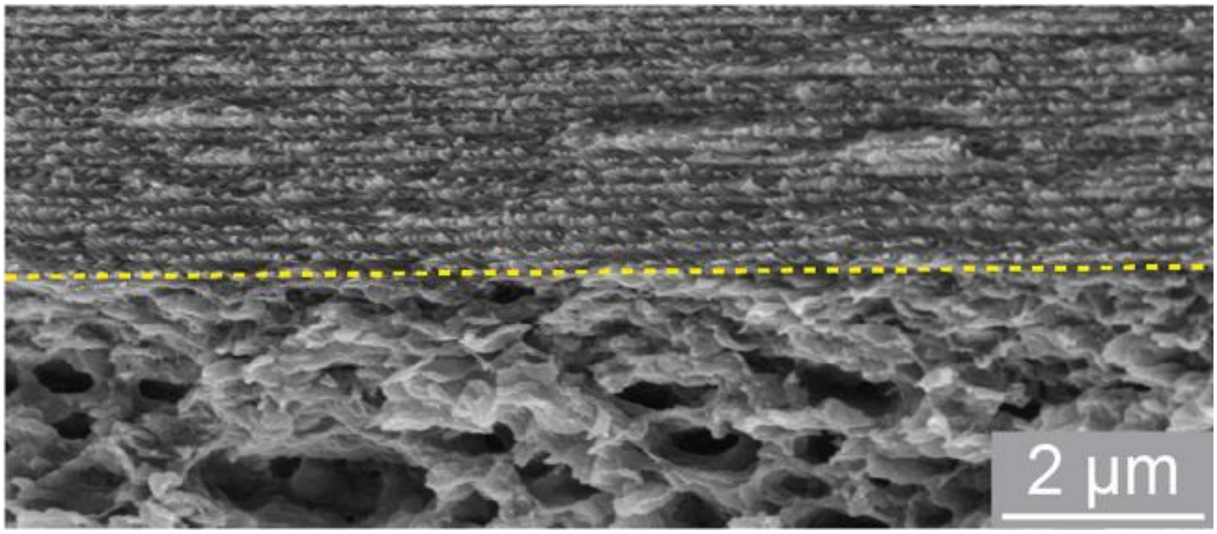

Fig. S12. Cross-sectional SEM image of the CNC-EC bilayer film, showing the clearly defined interface between the two nanostructures (top: CNC, bottom: EC).

(a)

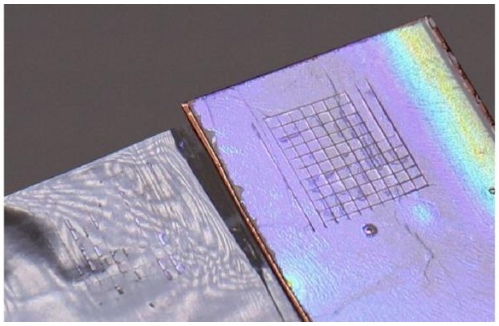

(b)

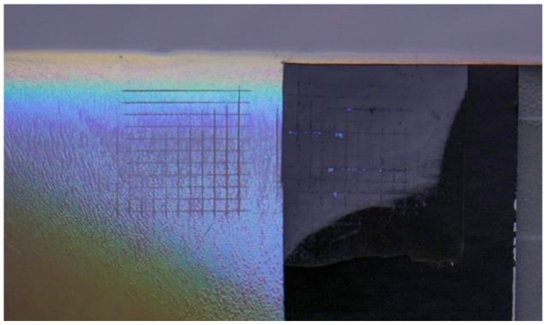

Fig. S13. Results of cross hatch adhesion test of **(a)** blue CNC-EC on a copper sheet with peeled tape on its left and **(b)** a free-standing blue CNC-EC bilayer films with peeled tape on its right. The structurally colored parts are the samples that underwent the cross hatch test.

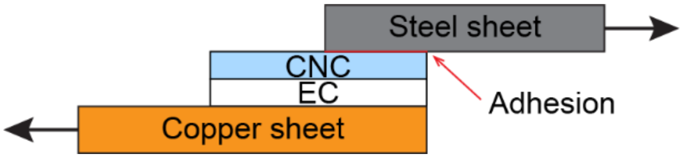

Fig. S14. Schematic of shear stress test for blue CNC-EC on a copper sheet.

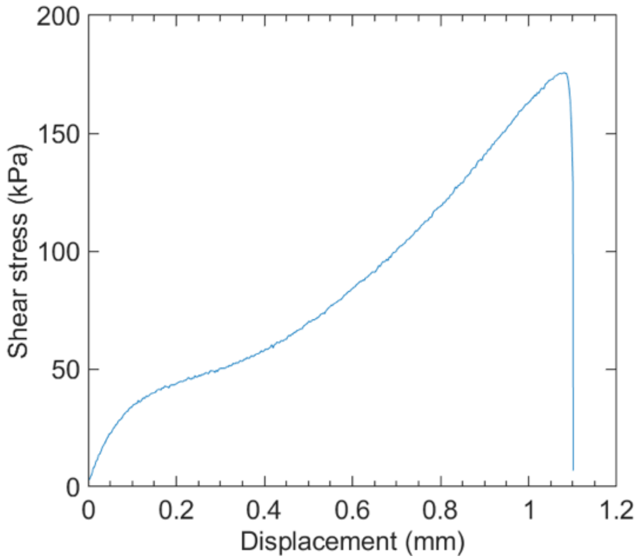

Fig. S15. Shear stress vs. displacement for blue CNC-EC on a copper sheet.

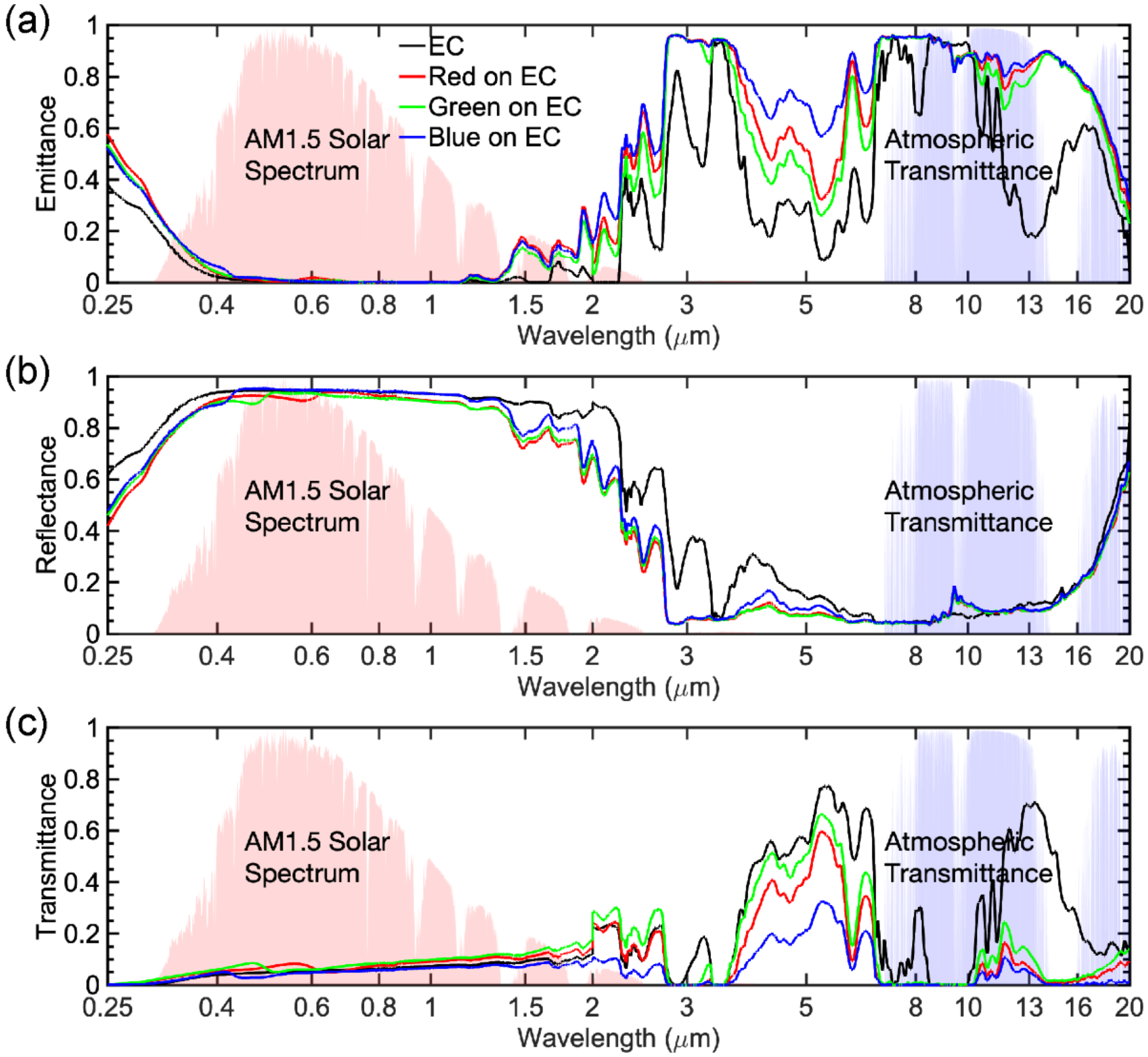

Fig. S16. (a) Emittance, **(b)** reflectance, and **(c)** transmittance of the EC film and CNC-EC bilayer films in the UV-VIS-IR spectral range.

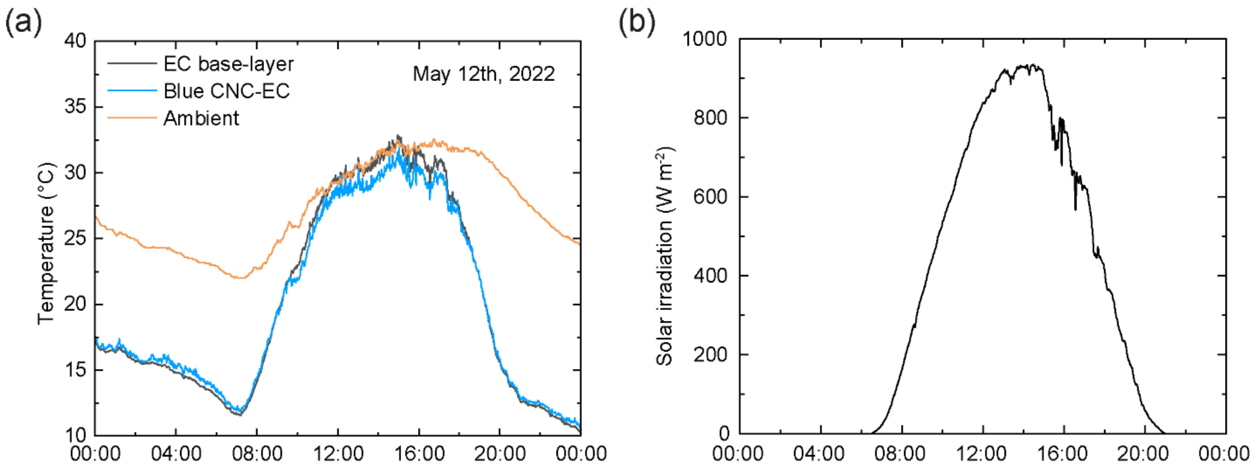

Fig. S17. (a) Raw temperature data and **(b)** solar irradiation during the 24-hour field test on May 12th, 2022.

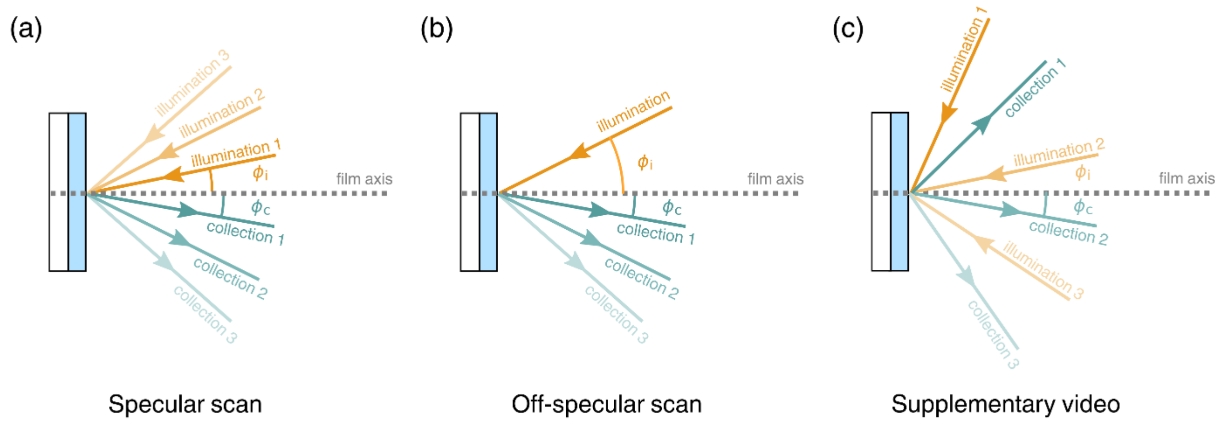

Fig. S18. Schematic of the illumination and collection procedures for angle-resolved spectroscopy (also referred to as goniometry), shown relative to a fixed frame of reference for the film. **(a)** Procedure for specular scans, with matched illumination and collection angles ($\Phi_i = \Phi_c$). **(b)** Procedure for off-specular scans, with fixed illumination angle (Φ_i) and varying collection angles (Φ_c). **(c)** Procedure for the Supplementary Video, where the overall angle between illumination and collection was kept fixed while the film was rotated.

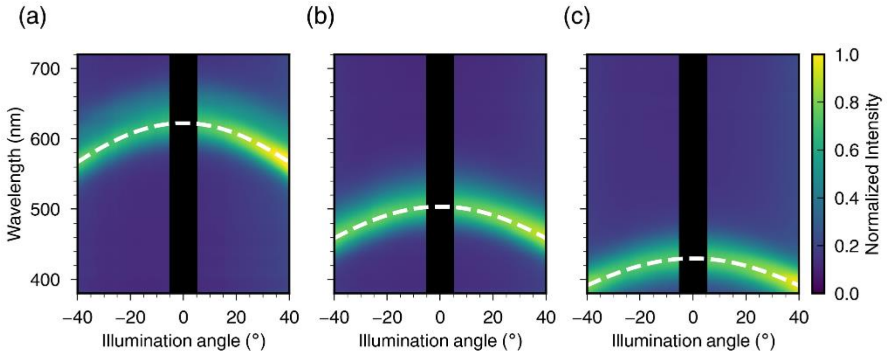

Fig. S19. Specular scans of **(a)** red, **(b)** green, and **(c)** blue CNC-EC bilayer films. The specular reflection from the CNC-EC bilayer film was measured by symmetrically increasing the angles of illumination and collection revealing a single peak at each angle, with a blue-shift at higher angles that is well described by Fergason's law (a modified version of Bragg's law of diffraction).[2]

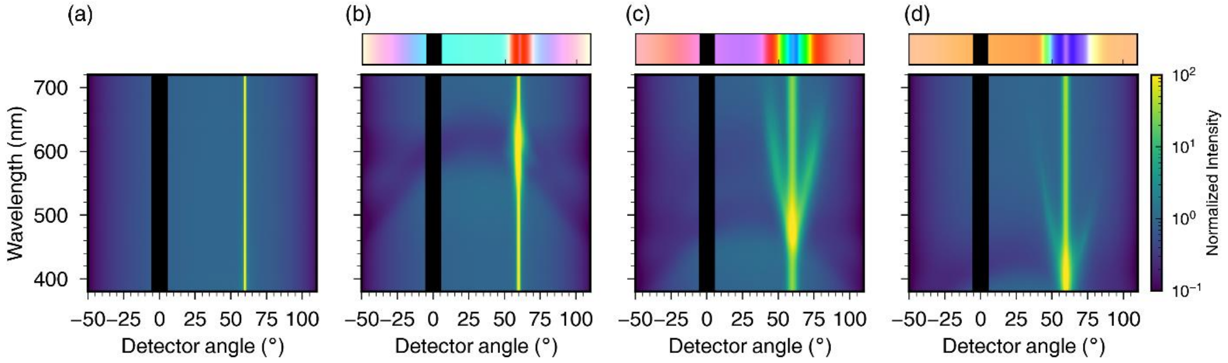

Fig. S20. Off-specular scans of **(a)** an EC film, and **(b)** red, **(c)** green, **(d)** blue CNC-EC bilayer films with the illumination angle fixed at 30° relative to the film normal. Color bars above each CNC-EC scan indicate the RGB color expected for the spectrum at each angle. At the specular angle (60°) the CNC reflection peak is observed (blue-shifted slightly due to Fergason's law). At most off-specular angles, the complementary subtractive color is observed. Additional spectral features are explained in the schematic in Fig. S22.

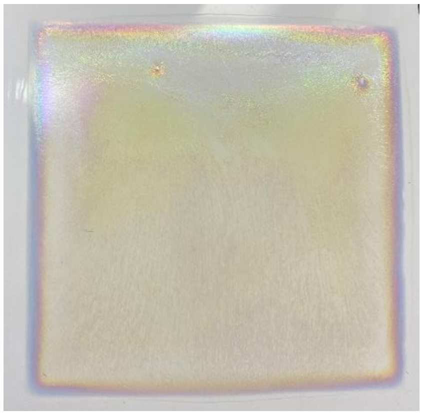

Fig. S21. Photograph of the blue CNC-EC bilayer film showing a yellowish color at off-specular angles.

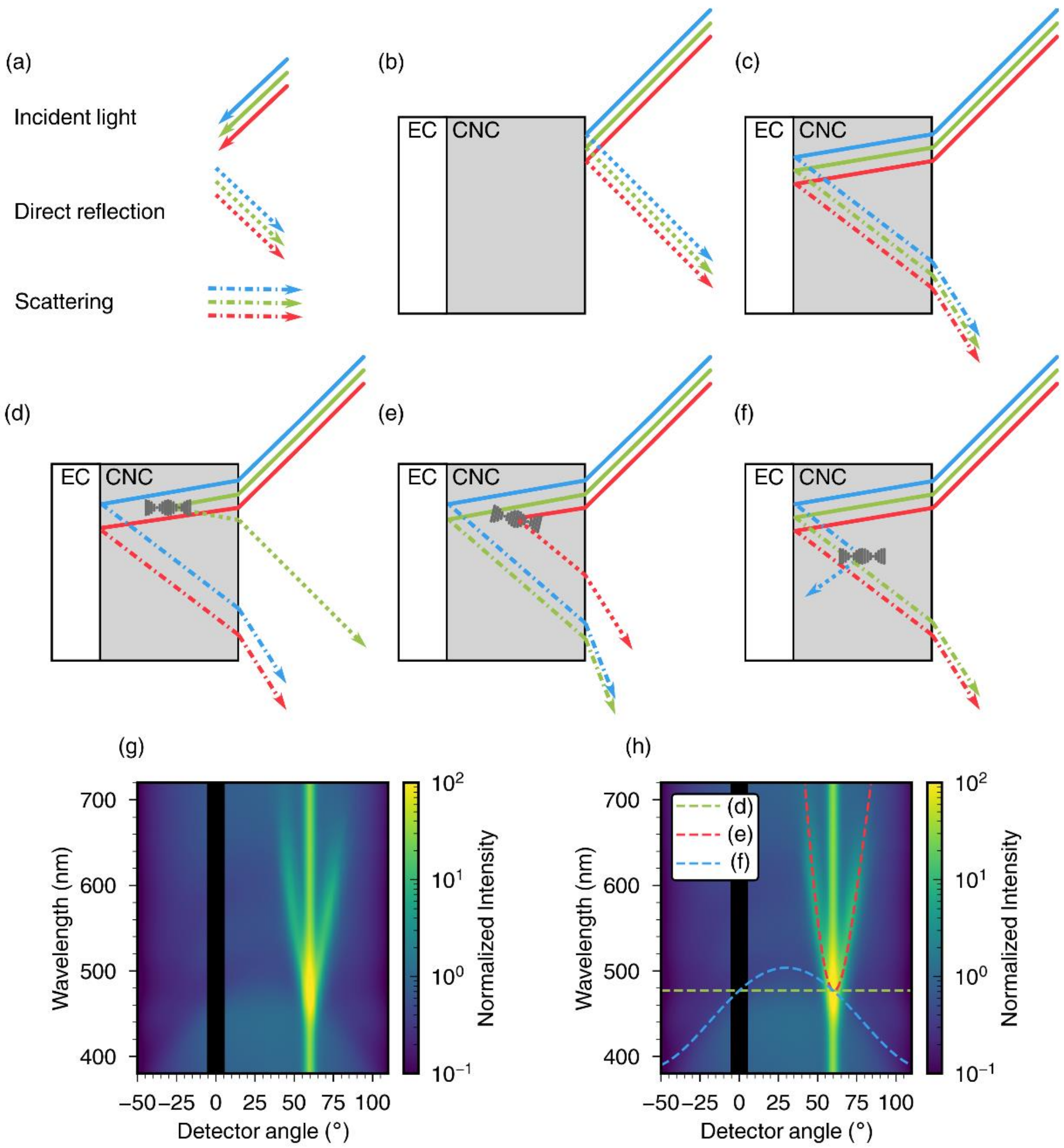

Fig. S22. Schematic illustrating various contributions to the angle-dependent optical response of EC-CNC films. **(a)** Legend indicating incident light, direct reflection and scattered light. **(b)** Direct reflection from the CNC-air interface. **(c)** Diffusive reflection from the EC layer after refraction through the CNC layer. **(d)** Wavelength-selective reflection from aligned CNC cholesteric domains **(e)** Red-shifted reflection from tilted and distorted CNC cholesteric domains.[3] **(f)** Back-reflection from the CNC cholesteric structure after initial diffusive reflection off the EC layer. **(g)** Off-specular angle-resolved spectroscopy scan for a green EC-CNC film (reproduced from Fig. S20 for ease of comparison). **(h)** Same as (g), but with dotted lines overlaid, indicating the effects due to the mechanisms shown in (d-f).

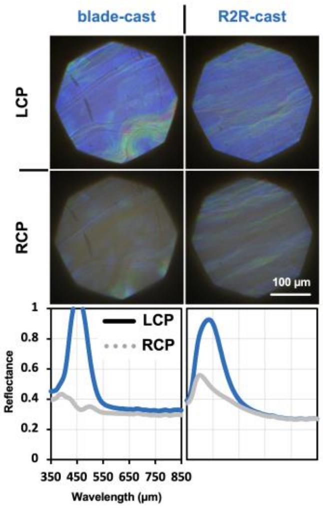

Fig. S23. Polarized optical microscope images and corresponding micro-spectra, recorded through left- and right- circularity polarized filters (denoted LCP and RCP), for the laboratory-scale blade-cast CNC film and the R2R-cast CNC-EC bilayer film. For the blade-cast CNC film, the reported spectrum is the average of at least 10 measurements while for the R2R-cast film, the reported spectrum is the average of at least 10 measurements on every 4 different locations along the R2R sample. The peak exceeding 1 is due to the back scattering from the EC layer adding to the reflection peak from the CNC layer.

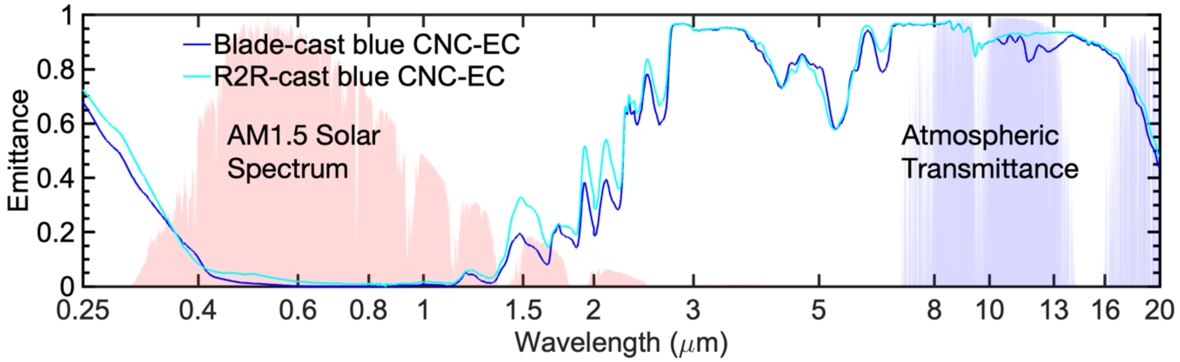

Fig. S24. Hemispheric emittance of the blue CNC-EC bilayer films prepared on laboratory-scale and large-scale from 250 nm to 20 μm. For the blade-cast and R2R-cast samples, their solar absorption and IR emittance across the transparent window have a difference of less than 2.4%.

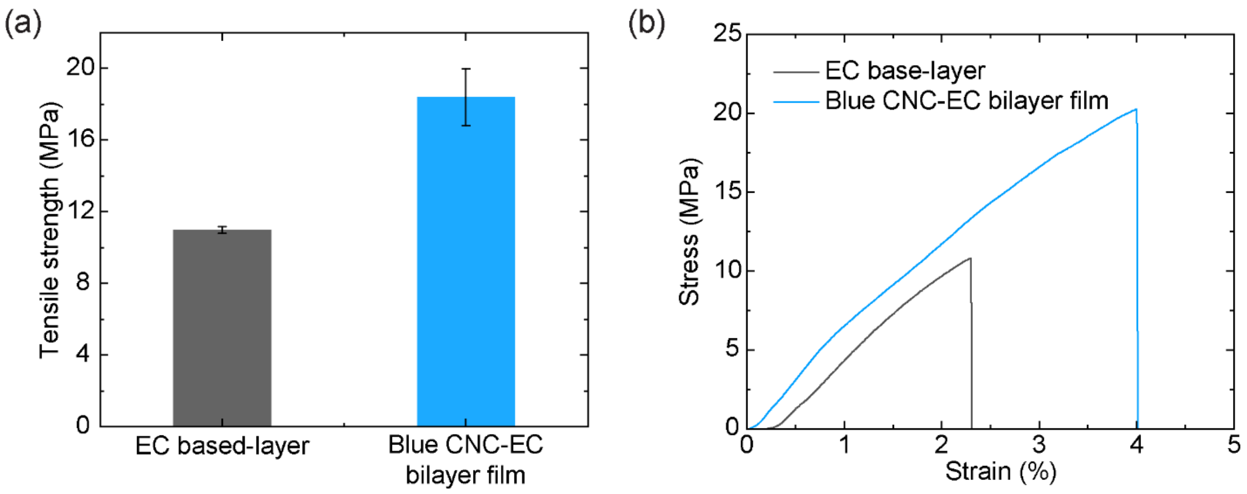

Fig. S25. (a) Averaged tensile strength and **(b)** strain-stress curves of blue CNC-EC bilayer films and EC base-layer.

References

[1] A. V. Tikhonravov, M. K. Trubetskov, G. W. DeBell, *Applied optics* **1996**, 35, 5493.
[2] J. L. Fergason, *Molecular Crystals and Liquid Crystals* **1966**, 1, 293.
[3] B. Frka-Petesic, G. Kamita, G. Guidetti, S. Vignolini, *Physical review materials* **2019**, 3, 045601.
